# Performance of emergency physicians utilizing a video-assisted semi-rigid fiberoptic stylet for intubation of a difficult airway in a high-fidelity simulated patient: a pilot study

**DOI:** 10.1186/1865-1380-5-24

**Published:** 2012-05-29

**Authors:** Derek R Cooney, Norma L Cooney, Harry Wallus, Susan Wojcik

**Affiliations:** 1 Department of Emergency Medicine, SUNY Upstate Medical University, 550 East Genesee / EMSTAT Center, Syracuse, NY, 13202, USA; 2 Department of Emergency Medicine, SUNY Upstate Medical University, 750 East Adams Street, Syracuse, NY, 13210, USA

**Keywords:** Airway, Clarus Video System, difficult, Emergency, Fiberoptic, Laryngoscopy, Levitan, Optical, Shikani, Stylet, StyletScope, Video

## Abstract

**Background:**

This study was designed to evaluate emergency physician success and satisfaction using a video-assisted semi-rigid fiberoptic stylet, the Clarus Video System (CVS), during a simulated difficult airway scenario.

**Findings:**

Emergency physicians (EPs) of all levels were first shown a brief slide show and three example videos, and then given 20 min to practice intubating a mannequin using both the CVS and standard direct laryngoscopy (DL). The mannequin was then placed in a c-collar and set to simulate an apneic patient with an edematous tongue and trismus. Each EP was given up to three timed attempts with each technique. They rated their satisfaction with the CVS, usefulness for their practice, and the effectiveness of the tutorial. Direct laryngoscopy had a 65% success rate on the first attempt, 20% on the second, and 15% required three or more. The CVS had a 100% success rate with a single attempt. Average time for independent DL attempts was 43.41 s (SD = ±26.82) and 38.71 s (SD = ±34.14) with CVS. Cumulative attempt times were analyzed and compared (DL = 74.55 ± 68.40 s and CVS = 38.71 ± 34.14 s; *p* = 0.028). EPs rated their satisfaction with, and usefulness of, the CVS as ≥6 out of 10.

**Conclusion:**

Emergency physicians were able to successfully intubate a simulated difficult airway model on the first attempt 100% of the time. Emergency physicians were satisfied with the CVS and felt that it would be useful in their practice.

## Findings

### Background

Emergency physicians may find that a clinical encounter with a difficult airway scenario is not only unexpected and unavoidable, but they may also find themselves forced to utilize a device with which they have little familiarity [1,2]. Among the different difficult airway devices available to EPs, there is a group of devices known as semi-rigid fiberoptic (optical) stylets [3-6]. There have been a number of reported successful intubations with this type of device [3,7-9] in patients with difficult airways and also in a number of simulated difficult airway scenarios [5,10-13].

**Figure 1:**
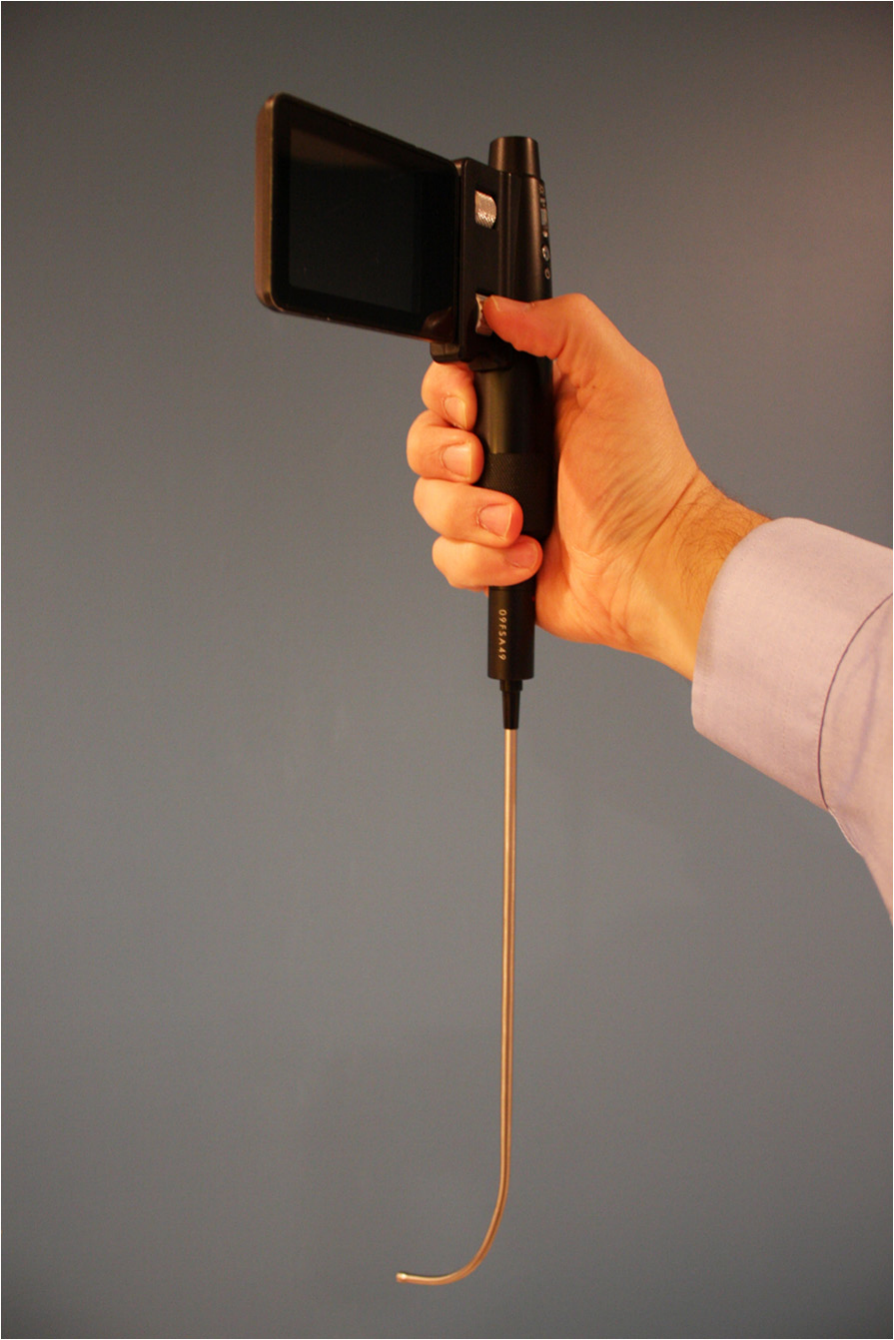
The Clarus Video System (Clarus Medical, Minneapolis, MN).

**Figure 2:**
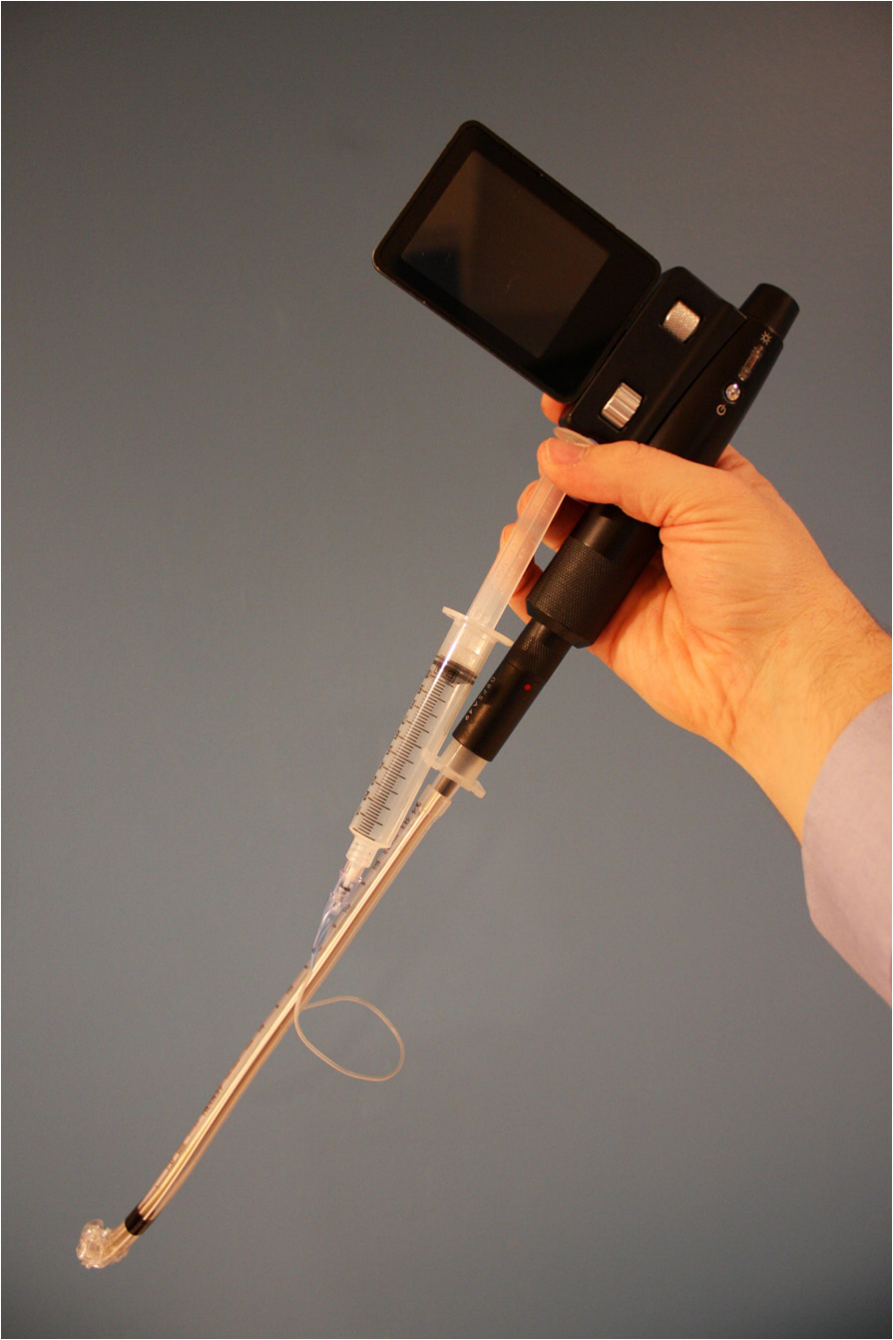
Endotracheal tube in position over the CVS.

The Clarus Video System is a video-assisted semi-rigid fiberoptic stylet that displays a view of the airway on a video screen attached to the side of the CVS (Figure 1). The endotracheal tube is loaded on the short semi-rigid malleable metal stylet with a fiberoptic distal light source just inside the tip of the tube (Figure 2). This study was designed to evaluate EP success and satisfaction using the Clarus Video System (CVS) during a simulated difficult airway scenario, with only a brief introductory tutorial on the operation of the device. Because the participants do not utilize this device in their current practice, this design allowed the authors to simulate a scenario that would illustrate the performance of emergency physicians with the CVS in an unexpected difficult airway situation with little to no experience with the device.

### Hypotheses

(1) Participants will achieve successful intubation in fewer attempts when utilizing the CVS. (2) The time for each intubation attempt will be less when utilizing the CVS. (3) The sum of the time for intubation attempts will be less when using the CVS.

## Methods

**Figure 3:**
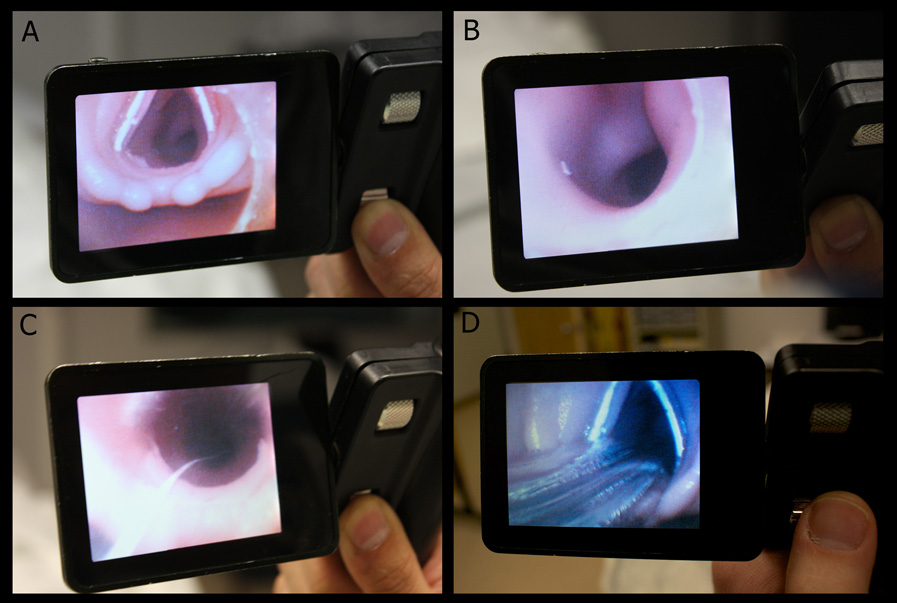
**Views as seen on the CVS screen: A.** View of the larynx of the mannequin with ETT loaded on the CVS. **B.** View of the mannequin’s trachea after positioning the end of the stylet through the vocal cords. **C.** View of the ETT passing through the cords as it is advanced off of the CVS. **D.** Confirmation of ETT placement by viewing the larynx with the CVS.

Attending and resident emergency physicians of all levels were first shown a brief slide show on the use of the CVS, followed by three example videos (http://clarus-medical.com/airway/products/cvs-clarus/videos). The slideshow was composed of 18 slides and was presented in approximately 10 min each time. Study participants were given 20 min to practice intubating the mannequin (Laerdal SimMan®, Wappingers Falls, NY, USA) in a group with a standard 7.5 endotracheal tube (Rusch: Teleflex Medical, Research Triangle Park, NC, USA), using both the CVS and standard direct laryngoscopy (DL). Instruction on utilizing the CVS alone (without the use of a laryngoscope) was given describing a midline approach with the device. Alternative approaches to the use of the CVS were not discussed. During the practice period the mannequin was set to simulate an apneic patient with normal airway, and no restrictions were imposed upon technique or positioning of the head or neck. For the data collection phase of the study, the mannequin was then placed in a c-collar and set to simulate an apneic patient with an edematous tongue, posterior pharyngeal swelling, and trismus. Random selection was used to determine which technique would be used first by each participant. Each EP was given up to three attempts to intubate with each technique. The attempts were timed from picking up the device until the stylet (either the CVS or a standard malleable stylet) was removed from the endotracheal tube (ETT) in the mannequin. Location of the ETT after each attempt was confirmed utilizing the CVS to visualize the larynx after both techniques (Figure 3). When the EP was successful with either technique, they then utilized the other technique. If unsuccessful after three attempts with either device, the EP was instructed to move to the other technique. After completion of the intubation attempts, EPs were asked to complete a demographic and survey form. They rated their satisfaction with the CVS, usefulness for their practice, and the effectiveness of the tutorial, with 0 = not at all and 10 = completely. Data were entered into SPSS® Statistics 19 (IBM®) and analyzed to determine the results for each technique. Analysis was done to evaluate for demographical associations (Table 1).

**Table 1:** Participant demographics

**Demographic information**				
Training Year:	1^st^ year = 6 (30%)	2^nd^ year = 4 (20%)	3^rd^ year = 3 (15%)	Attending = 7 (35%)
Handedness:	right = 17(85%)	left = 3(15%)	ambidextrous = 0(0%)	
Eye dominance:	right = 11(55%)	left = 2(10%)	unknown = 7(35%)	
Age Range:	25 yo–43 yo (mean = 33 yo)			
Sex:	male = 16 (80%) / female = 4 (20%)			
**Previous experience with difficult airway techniques (number of participants)**				
	none	simulated patient	actual patient	
Claurs Video Scope:	20 (100%)	0 (0%)	0 (0%)	
other optical stylet:	14 (70%)	5 (25%)	2 (10%)	
flexible fiberscope:	14 (70%)	3 (15%)	3 (15%)	
Glidescope:	3 (15%)	7 (35%)	16 (80%)	
other video-laryngoscope:	16 (80%)	3 (15%)	2 (10%)	
Airtraq:	17 (85%)	2 (10%)	0 (0%)	
lighted stylet:	16 (80%)	3 (15%)	1 (5%)	
gum elastic bougie:	4 (20%)	9 (45%)	13 (65%)	

## Results

Participant demographics and previous experiences with difficult airway devices are included in Table 1. During timed intubation attempts, when utilizing DL participants had a 65% success rate on the first attempt, 20% were successful on the second attempt, and 15% required three or more attempts. One EP failed to intubate with DL after three attempts. When utilizing the CVS, participants had a 100% success rate with a single attempt. There was a statistically significant difference in the number of attempts favoring the CVS (*p* = 0.012). Results of the time trials are detailed in Table 2. Average time for each DL attempt was 43.41 s (SD = ±26.82). Average time for each attempt with the CVS was 38.71 s (SD = ±34.14), but there was no significant difference between the two techniques (*p* = 0.270), and therefore the second hypothesis was incorrect. When the total time of the attempts until successful intubation was analyzed and compared, the mean total time of cumulative attempts for DL was 74.55 (SD = ±68.40) s, and for the CVS the mean total was 38.71 (SD = ±34.14) s. This difference was statistically significant favoring the CVS (*p* = 0.028). These results are summarized in Table 3. No significant differences were noted among participants in different training levels or in relation to reported handedness or eye dominance. All EPs rated their satisfaction with, and usefulness of, the CVS as ≥6 out of 10. Almost half (47.4%) were very satisfied (9-10/10). The effectiveness of the tutorial was rated as 9-10/10 by 57.9%.

**Table 2:** Individual results for EP intubation attempts by technique

Time in s. listed by participant. (*successful attempt)						
	DL attempt #1	DL attempt #2	DL attempt #3	CVS attempt #1	CVS attempt #2	CVS attempt #3
1	118.5	74.6*	n/a	102.3*	n/a	n/a
2	70.9	59.4	43.8*	27.7*	n/a	n/a
3	39.8*	n/a	n/a	134.8*	n/a	n/a
4	43.1*	n/a	n/a	15*	n/a	n/a
5	15.6*	n/a	n/a	11.7*	n/a	n/a
6	82.4	39.9*	n/a	16.7*	n/a	n/a
7	13*	n/a	n/a	8.2*	n/a	n/a
8	15.9*	n/a	n/a	21*	n/a	n/a
9	100.6	58	25.4	86.8*	n/a	n/a
10	92.4	79.7	46*	26.6*	n/a	n/a
11	34.1	62.3*	n/a	50.5*	n/a	n/a
12	106*	n/a	n/a	20.9*	n/a	n/a
13	22.3*	n/a	n/a	26.4*	n/a	n/a
14	35.7*	n/a	n/a	16.5*	n/a	n/a
15	20.6*	n/a	n/a	60.8*	n/a	n/a
16	17.5*	n/a	n/a	14*	n/a	n/a
17	58.3*	n/a	n/a	26.3*	n/a	n/a
18	24.7*	n/a	n/a	25.1*	n/a	n/a
19	29.3	36.4*	n/a	22.5*	n/a	n/a
20	24.8*	n/a	n/a	60.3*	n/a	n/a

**Table 3:** Mean number of intubation attempts and times for intubation attempts (per attempt and cumulative)

**(Mean time to intubation in s)**			
	Direct laryngoscopy (DL)	Clarus Video System(CVS)	
Mean number of attempts per provider:	1.5 (SD = ±0.76)	1 (SD = ±0)	*p* = 0.012
Mean time for each attempt:	43.41 (SD = ±26.82)	38.71 (SD = ±34.14)	*p* = 0.270
Mean time for all attempts:	74.55 (SD = ±68.40)	38.71 (SD = ±34.14)	*p* = 0.028

### Current study limitations

Simulated patient encounters, even when a high-fidelity simulator is employed, are not equal to live patient encounters. However, this study could not be performed in the real-time clinical environment because of the need to study difficult airway management in a reproducible way, and without any potential for patient harm due to a lack of provider expertise with the studied device. This investigation was conducted in a small sample at a single center in participants that were being observed by attending physicians with whom they were familiar. Some concern for a possible Hawthorne effect could be expressed. This study does not compare the CVS to other potentially available difficult airway intubation devices. Based on these limitations some caution should be advised when attempting to generalize these findings. Further study in a larger, more diverse sample is needed to strengthen these conclusions.

## Discussion

Other studies have compared fiberoptic stylets to other difficult airway techniques and found favorable results in simulated difficult airway models [12,13]. When studied in intubations on live volunteers wearing cervical collars to limit s-spine mobility, a fiberoptic stylet of a slightly different design also had favorable results [9,11].

## Conclusions

Emergency physicians, with no previous experience with the device and only a brief introductory tutorial, were able to successfully intubate a simulated difficult airway model on the first attempt 100% of the time utilizing a video-assisted semi-rigid fiberoptic stylet. In this pilot study, the Clarus Video System was superior overall to direct laryngoscopy. This conclusion is supported by the 100% first attempt success rate (compared to 65% for DL) and the fact that the CVS had a lower cumulative attempt time. The majority of EPs was satisfied with the CVS and felt that it would be useful in their practices. Familiarity with the CVS may decrease times, and further study of this, as well as comparisons to other types of difficult airway intubation devices, utilizing this experimental model is appropriate. Future study of this model in a larger, more diverse sample is indicated.

## Abbreviations

CVS, Clarus Video System; EP, emergency physician; DL, direct laryngoscopy; ETT, endotracheal tube.

## Competing interests

The authors declare that they have no competing interests and specifically have no personal or financial relationship with Clarus Medical or any of its affiliates. No funds or honoraria were received in the support of this study. No editorial or statistical assistance or rights were extended to, or claimed by, the manufacturer, and none were received. A single loaner device was provided by Clarus Medical at no cost to the investigators. No “ghost writing” occurred or was considered. In addition, all images, figures, and tables are original work of the authors.

## Authors’ contributions

Drs. Cooney designed the study and, along with Dr. Wallus, carried out data collection and prepared the manuscript. Dr. Wojcik provided statistical analysis and editorial support services to the project. All photos and tables are by Dr. D. Cooney.

## Authors’ information

DRC is the Director of Prehospital Medicine and the Program Director for the EMS & Disaster Medicine Fellowship at SUNY Upstate Medical University. NLC is an Assistant Professor of Emergency Medicine and the Medical Director of Emergency Services at Oswego Hospital, a community hospital in Oswego, New York. HW is a Clinical Instructor of Emergency Medicine and completing his EMS & Disaster Medicine Fellowship training at the time of submission of this manuscript. SW is an Assistant Professor (PhD) and a member of the research faculty of the Department of Emergency Medicine at SUNY Upstate Medical University.

## References

[B1] Levitan RM Design rationale and intended use of a short optical stylet for routine fiberoptic augmentation of emergency laryngoscopy Am J Emerg Med 2006 Jul 24 4 490 495 10.1016/j.ajem.2005.12.024 16787811

[B2] Levitan RM Everett WW Ochroch AE Limitations of difficult airway prediction in patients intubated in the emergency department Ann Emerg Med 2004 44 307 313 10.1016/j.annemergmed.2004.05.006 15459613

[B3] Bein B Worthmann F Scholz J Brinkmann F Tonner PH Steinfath M Dörges V A comparison of the intubating laryngeal mask airway and the Bonfils intubation fibrescope in patients with predicted difficult airways Anaesthesia 2004 Jul 59 7 668 674 10.1111/j.1365-2044.2004.03778.x 15200542

[B4] Liem EB Bjoraker DG Gravenstein D New options for airway management: intubating fibreoptic stylets Br J Anaesth 2003 Sep 91 3 408 418 10.1093/bja/aeg011 12925482

[B5] Biro P Weiss M Gerber A Pasch T Comparison of a new video-optical intubation stylet versus the conventional malleable stylet in simulated difficult tracheal intubation Anaesthesia 2000 Sep 55 9 886 889 10.1046/j.1365-2044.2000.01519.x 10947753

[B6] Shikani AH New “seeing” stylet-scope and method for the management of the difficult airway Otolaryngol Head Neck Surg 1999 Jan 120 1 113 116 10.1016/S0194-5998(99)70380-3 9914560

[B7] Kovacs G Law AJ Petrie D Awake fiberoptic intubation using an optical stylet in an anticipated difficult airway Ann Emerg Med 2007 Jan 49 1 81 83 10.1016/j.annemergmed.2006.03.024 17141147

[B8] Saus JA Initial clinical experience with the Clarus Video System in a morbidly obese patient in respiratory arrest 2010, http://clarus-medical.com/wp-content/files_mf/1276015538ClarusVideoSystemWhitePaper2010.pdf

[B9] Kim JK Kim JA Kim CS Ahn HJ Yang MK Choi SJ Comparison of tracheal intubation with the Airway Scope or Clarus Video System in patients with cervical collars Anaesthesia 2011 Aug 66 8 694 698 10.1111/j.1365-2044.2011.06762.x 21564045

[B10] Ong JR Chong CF Chen CC Wang TL Lin CM Chang SC Comparing the performance of traditional direct laryngoscope with three indirect laryngoscopes: A prospective manikin study in normal and difficult airway scenarios Emerg Med Australas 2011 Oct 23 5 606 614 10.1111/j.1742-6723.2011.01441.x 21995476

[B11] Komatsu R Kamata K Hamada K Sessler DI Ozaki M Airway scope and StyletScope for tracheal intubation in a simulated difficult airway Anesth Analg 2009 Jan 108 1 273 279 10.1213/ane.0b013e31818a4398 19095862

[B12] Kovacs G Law JA McCrossin C Vu M Leblanc D Gao J A comparison of a fiberoptic stylet and a bougie as adjuncts to direct laryngoscopy in a manikin-simulated difficult airway Ann Emerg Med Dec 50 6 676 685 Epub 2007 Aug 3 17681639 10.1016/j.annemergmed.2007.05.022

[B13] Evans A Morris S Petterson J Hall JE A comparison of the Seeing Optical Stylet and the gum elastic bougie in simulated difficult tracheal intubation: a manikin study Anaesthesia 2006 May 61 5 478 481 10.1111/j.1365-2044.2006.04539.x 16674624

